# RAZOR: A Compression and Classification Solution for the Internet of Things

**DOI:** 10.3390/s140100068

**Published:** 2013-12-19

**Authors:** Matteo Danieletto, Nicola Bui, Michele Zorzi

**Affiliations:** 1 Department of Information Engineering (DEI), University of Padova, Via Gradenigo, n.6b, 35131 Padova, Italy; E-Mail: zorzi@dei.unipd.it; 2 Institute IMDEA Networks, Av. del Mar Mediterraneo, 22, 28918 Leganes, Madrid, Spain; E-Mail: nicola.bui@imdea.org; 3 Consorzio Ferrara Ricerche, Via Saragat, 1-Block B-1st Floor, 44122 Ferrara, Italy

**Keywords:** signal processing, motif, compression, classification, computational complexity, Internet of Things

## Abstract

The Internet of Things is expected to increase the amount of data produced and exchanged in the network, due to the huge number of smart objects that will interact with one another. The related information management and transmission costs are increasing and becoming an almost unbearable burden, due to the unprecedented number of data sources and the intrinsic vastness and variety of the datasets. In this paper, we propose RAZOR, a novel lightweight algorithm for data compression and classification, which is expected to alleviate both aspects by leveraging the advantages offered by data mining methods for optimizing communications and by enhancing information transmission to simplify data classification. In particular, RAZOR leverages the concept of motifs, recurrent features used for signal categorization, in order to compress data streams: in such a way, it is possible to achieve compression levels of up to an order of magnitude, while maintaining the signal distortion within acceptable bounds and allowing for simple lightweight distributed classification. In addition, RAZOR is designed to keep the computational complexity low, in order to allow its implementation in the most constrained devices. The paper provides results about the algorithm configuration and a performance comparison against state-of-the-art signal processing techniques.

## 1. Introduction

In this work, we propose a lightweight algorithm, named RAZOR, for data compression and classification for the Internet of Things (IoT) [1] that consists of the integration of smart connected objects into the standard Internet. In this paper we call “smart” those devices that, though hardware constrained, are still capable of performing their task by exploiting their limited capabilities in clever ways.

RAZOR is developed to exploit historical data information to:

1. Compress IoT data using recurrent patterns of a specific physical phenomenon.
2. Classify IoT data according to the occurrence of the same recurrent patterns.

As a further motivation, we considered these characteristics of the IoT: (1) data is measured by a huge number of (mostly) constrained devices; (2) a variety of physical phenomena, often incommensurable with one another, are monitored (typical information exchanged in the IoT includes simple readings (e.g., temperature, light and humidity), and simple operational states (e.g., energy consumption, production chain triggers and presence detection)); (3) recurrent data patterns can be found in the temporal and spatial evolution of the data (as an example, similar outputs are likely to be gathered by closely deployed devices and to be repeated over the same hours of different days). As a concrete application example, Wireless Sensor and Actuator Networks (WSAN) [2] capabilities will be our reference for the rest of the paper, and in particular, we will take those of the telosb architecture [3] as representative for the whole category, *i.e*., 1 MHz CPU and a 250 kbps nominal data rate.

In our previous work [4], we demonstrated that *motifs* [5], recurrent patterns of a given time series, can be used to improve communications and to classify data. In this paper, we further optimize our technique and provide a thorough performance evaluation, including a comparison with state-of-the-art compression and classification solutions. We also discuss the computational complexity of the RAZOR procedures and its impact on the energy consumption of constrained devices. Finally, in addition to a thorough analysis on synthetic datasets, we test our solution against real data collected in an actual WSAN deployment measuring temperature, humidity and light in a public building.

The main contributions of the paper are the following:

1. We develop a lightweight data compressor and classificator, called RAZOR;
2. We show that RAZOR is able to achieve satisfactory results in terms of both compression and classification when tested on both synthetic and real data;
3. We show that RAZOR can be successfully implemented on constrained devices (e.g., wireless sensor nodes), as it does not require a large amount of memory, nor a powerful processor;
4. We study RAZOR's applicability to multi-hop and lossy environments.

The rest of the paper is organized as follows: Section 2 illustrates RAZOR's inspiration sources and the state-of-the-art algorithms we took as benchmarks; Section 3 provides a high-level system description by illustrating the different modules, their functionalities and their interactions; Section 4 describes RAZOR and its evolution from our previous algorithm, highlighting the roles of the many parameters and variants; Section 5 reports the results obtained in our evaluation campaign providing insight on the best parameter selection, the performance comparison and the results on the real scenario; finally, Section 7 concludes the paper.

## 2. Related Work

First of all, our work would not have been possible without the fundamental contributions by Gersho and Gray on vector quantization [6], Keogh and his group on data mining [7] and Bishop on pattern recognition [8].

Vector quantization techniques are the most similar to our algorithm, since they are based on the creation of a Codebook from a training dataset and its subsequent usage for replacing new segments of the time series with one element from the Codebook. Furthermore, Murakami in [9] developed a technique that stores normalized signals in the Codebook, a concept similar to our solution (see Section 4).

Bishop's pattern recognition book [8] is one of the most comprehensive works on data patterns, considering both parametric and non-parametric models. In addition, we considered the data mining techniques developed by Keogh's group in [5,10–12] for comparison to our algorithm.

In particular, [12] studies a parameter free data mining technique using a Compression-based Dissimilarity Measure (CDM), which proves to be very efficient for classification, while [10,11] use a Symbolic Aggregate approXimation (SAX) technique for finding Motifs in a given time series. However, all these techniques are either too computationally complex (the former) or too lossy (the latter) with respect to our requirements. In addition, while they concentrate on exploiting motifs for signal classification, we need to use them for data compression as well.

Another important milestone is [13], which proposes a clustering technique based on compression and provides a rigorous analytical framework based on the notion of Kolmogorov complexity.

Several other algorithms to combine compression and communication can be found in the literature: the reader is referred to [14] for a detailed survey on in-network aggregation techniques and to [15] for studies on Compressive Sensing. Reference [16] studies time series compression and prediction quality in WSANs. However, all these techniques, while being very efficient at compressing information, do not provide any means to recognize and classify data sources. In addition, [17] provides one of the latest compendia on compression algorithms for constrained devices. Finally, the techniques we chose as a benchmark for RAZOR are: (1) lossy compression through the Discrete Cosine Transform (DCT) [18]; (2) Lightweight Temporal Compression (LTC) [19]; and (3) Enumeration of Motifs through Matrix Approximation (EMMA) [5]. The motivations for such a selection are the popularity of DCT for lossy compression in audio and video application, the effectiveness, while maintaining a low computational complexity, of LTC and the efficiency of EMMA in classifying many different signal types.

Furthermore, exploiting the parallelism between bi-dimensional data maps and images, Wang [20] proposed the application of image compression techniques to WSANs. Starting from this idea and using Low Energy Adaptive Clustering Hierarchy (LEACH) [21] to partition the network into clusters, the cluster-based and robust information-driven architecture (RIDA [22]) is proposed to apply compression algorithms, such as DCT or Wagner-Discrete Wavelet Transform (Wagner-DWT), to logical maps of data collected within clusters in order to improve compression performance, communication efficiency and robustness.

## 3. System Overview

The networking community uses the term IoT with several different meanings. However, all the different opinions agree on the need for smart-objects to be connected through the Internet. This objective can be achieved in several ways and with different technologies, such as Radio Frequency Identifier (RFID), Wireless Sensor Networks (WSAN), *etc*. Each solution can adopt a different communication paradigm, such as ZigBee or IPv6 over Low power Wireless Personal Area Networks (6LoWPAN), and we refer to each sub-network adopting a homogeneous communication solution as an IoT island. The IoT has several goals, and one of them is the optimization of data exchange through strategic resource preservation, *i.e*., saving battery charge and reducing communication overhead. As a concrete example of a typical IoT island, we focus on a single WSAN composed of many constrained devices, referred to as *nodes*, characterized by sensing and/or actuation capabilities, limited resources (∼10 kB RAM, ∼50 kB ROM, battery powered) and computational capabilities (1 MHz CPU) and low data rate (≤ 250 kb/s). Usually, these nodes need to use more powerful devices, referred to as *gateways*, to be connected to the Internet and to be able to be interacted with. The reader is referred to [2] for details about constrained node communication and Internetworking.

**Figure 1.**
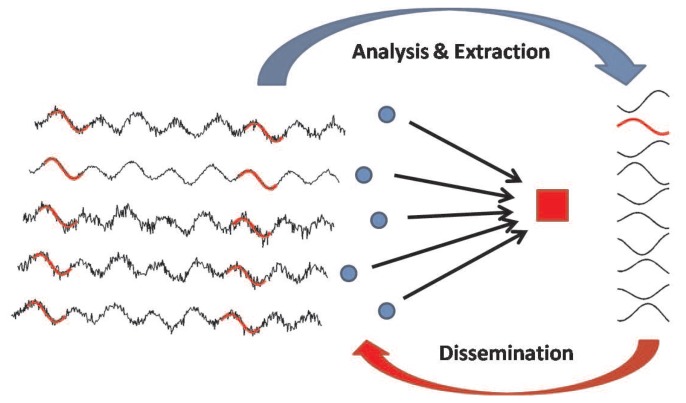
Schematic diagram of a simple Internet of Things (IoT) network: blue circles and the red square represent sensor nodes and a gateway node, respectively.

Figure 1 illustrates how the system works: sensor nodes (blue circles) produce information flows related to the same physical phenomenon and transmit the raw information towards a gateway. For simplicity, we assume that a single phenomenon is monitored and that all nodes are of the same type, and leave more complex scenarios for future work. The gateway, which is unaware of the particular type of information carried by the data flows, analyzes the raw data in order to identify the most frequent trends. After an initial training period, the gateway is able to compute a set of patterns and to disseminate them back to the nodes (details are in Section 4.1). Then, the nodes compare the sensed information with the received pattern set, and instead of sending the whole data sequence, they just send the pattern identifier (details are in Section 4.2).

## 4. The RAZOR Algorithm

In this section, we will describe the algorithms composing RAZOR: we will start from the extraction of the Motif Codebook, *B*, from a given training set of samples in Section 4.1, and then we will discuss the usage of this Codebook(s) for compressing and identifying the original signal in Section 4.2.

### 4.1. Motif Extraction

RAZOR is inspired by two different applications of signal processing for time series: on the one hand, our solution can be categorized as part of the vector quantization techniques [6], but on the other hand, we apply solutions derived from data mining and pattern recognition [8,10] during the learning phase. In order to understand the impact of the two inspiration sources, we will provide a mathematical definition of the problem as follows.

The original signal that we want to analyze is:

(1) S(t)∈ℝ,t∈[0,+∞)

but in order to maintain the signal as processable in a finite time, we will focus on a subset of *S*(*t*), called the *training set*, of length *M_S_* and defined as:

(2) $$T(t)=S(t),t\in [0,M_{S}]$$

Please note that the training set can be composed of the inputs from different sources, but we will discuss this case after the description of the basic concepts.

We assume that the signal and the training set are sampled with period *T*, respecting the Nyquist–Shannon sampling theorem, and quantized on an alphabet, 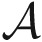 ⊂ ℝ, of *L* symbols:

(3) $$\begin{array}{ll} S_{i} & =Q(S(iT))\in A,\\ T_{i} & =S_{i},i=0,1,\ldots ,M-1 \end{array}$$

where *S_i_* and *T_i_* are samples of the original signal and of the training set, respectively, *M* = *M_S_*/*T* is the length of the sampled signal and *Q*(·) is an *L*-level quantization function. Notice that, while the sampling period, *T*, and the number of levels, *L*, are usually given by the application, it is also possible to adapt these two parameters dynamically; however, this opportunity is not covered in the current work. As in standard Vector Quantization, our objective in this section is to extract a Motif Codebook from the training set. The parameters governing this Codebook are three:

1. *K*, the number of motifs in the Codebook,
2. *N*, the length of the motifs in samples,
3. *b*, the number of bits used to encode each sample of the motifs in the Codebook.

In Section 5, we will analyze the impact of each of these parameters on the performance metrics. The first step of the extraction algorithm is to identify in the training set a number of segments of *N* samples each. Each segment is defined as:

(4) $$T^{\left(i\right)}=[T_{i},T_{i+1},\ldots ,T_{i+N-1}],i=0,1,\ldots ,M-N+1$$

representing a set of *N* consecutive samples of the training set starting from sample *T_i_*.

#### Segment normalization

In our previous paper [4], we analyzed two representation techniques for storing the motifs of the Codebook. In particular, we found that our *Normal Shapes* representation technique, aimed at obtaining motifs with zero mean and unit variance, were appropriate for our objectives of compression and classification for constrained devices. The idea of using normalized segments, instead of their absolute values, can also be found in standard Vector Quantization [9]; in such a way, the Codebook contains a set of templates that can be used as building blocks for the original signal after appropriate de-normalization.

Normal shapes are obtained from the original segments by subtracting the offset and dividing by an amplitude gain. The offset can be straightforwardly computed as the mean value of the segment under analysis; instead, although in [4] we only considered the standard deviation (STD) (as in [9]) as the amplitude gain, here, we will extend our investigation to two more quantities:

1. the square root of the segment energy (GAIN), as defined in [6]; and
2. the maximum deviation (MAXABS).

In the following, we will indicate the Normal Shape of a given segment, *S*^(^*^i^*^)^, as *S̅*^(^*^i^*^)^, which is obtained as follows:

(5) $${\bar{S}}^{\left(i\right)}=(S^{\left(i\right)}-O^{\left(i\right)})/G^{\left(i\right)}$$

where *O*^(^*^i^*^)^ is the offset of segment *S*^(^*^i^*^)^ and is computed as:

(6) $$O^{\left(i\right)}=(1/N)\sum_{k=0}^{N-1}S_{i+k}$$

and *G*^(^*^i^*^)^ is the amplitude gain of *S*^(^*^i^*^)^ and can assume one of the three following expressions:

(7) $$G^{\left(i\right)}=\{\begin{array}{ll} \sigma_{S^{\left(i\right)}} & \left(\text{STD}\right)\\ \sqrt{(\sum_{k=0}^{N-1}S_{i+k}^{2})/N} & \left(\text{GAIN}\right)\\ \underset{k\in [0,N-1]}{\max}(\left|S_{i+k}-O^{\left(i\right)}\right|) & \left(\text{MAXABS}\right) \end{array}$$

In the following, we will define the de-normalized version of a given segment or motif with the subscript, *R*. For instance, the de-normalized version of *S̅*^(^*^i^*^)^, will be:

(8) $$S_{R}^{\left(i\right)}={\bar{S}}^{\left(i\right)}G^{\left(i\right)}+O^{\left(i\right)}=S^{\left(i\right)}$$

which is the same as the original version if no additional quantization is used.

We will provide a numerical comparison among the three techniques in Section 5.

As for standard Vector Quantization techniques, we now need to compare all the normalized segment couples, *T̅*^(^*^i^*^)^ and *T̅*^(^*^j^*^)^, in terms of their relative distance:

(9) $$d({\bar{T}}^{\left(i\right)},{\bar{T}}^{\left(j\right)})\in \mathbb{R}$$

where *d*(·) is the adopted dissimilarity function.

#### Dissimilarity functions

In this paper, to compute the distance between two segments of a signal, we take into consideration the following dissimilarity functions: Euclidean distance, infinity norm, minimum distance (MINDIST) [10], Compression-based Dissimilarity Measure (CDM) [12] and DTW [23]. With respect to our previous work [4], we added the infinity norm, which has two interesting properties for RAZOR:

1. it provides an upper bound on the distance between two samples;
2. it is computationally simpler than the Euclidean distance.

We already dismissed MINDIST, since it is specific for piecewise aggregate approximation (PAA), CDM, since it is only efficient for large files or for a large number of samples, and DTW, since it is too computationally demanding for our reference hardware. Hence, in this work, our dissimilarity functions will be *L*2 and the infinity norm, and we will compare RAZOR's performance adopting these two distances in Section 5. Thus:

(10) $$d({\bar{T}}^{\left(i\right)},{\bar{T}}^{\left(j\right)})=\{\begin{array}{c} {\left\|{\bar{T}}^{\left(i\right)}-{\bar{T}}^{\left(j\right)}\right\|}_{2}\\ {\left\|{\bar{T}}^{\left(i\right)}-{\bar{T}}^{\left(j\right)}\right\|}_{\infty } \end{array}$$

where the two norms are applied as follows:

(11) $$\begin{array}{l} {\left\|{\bar{T}}^{\left(i\right)}-{\bar{T}}^{\left(j\right)}\right\|}_{2}=\sqrt{\sum_{k=0}^{N-1}{\left|{\bar{T}}_{i+k}-{\bar{T}}_{j+k}\right|}^{2}}\\ {\left\|{\bar{T}}^{\left(i\right)}-{\bar{T}}^{\left(j\right)}\right\|}_{\infty }=\max_{k\in [0,N-1]}(\left|{\bar{T}}_{i+k}-{\bar{T}}_{j+k}\right|) \end{array}$$

#### Codebook selection

In the following, we will summarize our Codebook selection algorithm highlighting the differences from the previous version. In [4], we analyzed two different algorithms for the selection of the motifs to create the Codebook. In particular, we found that our *Matching-Based* approach, a lightweight motif selection algorithm inspired by data mining applications [10], is appropriate for our objectives of compression and classification for constrained devices.

The basic idea for creating the Codebook is that of selecting those motifs that have the most frequent shape among the normalized segments in the training set. In order to run our algorithm, which is listed in Algorithm 1, we first need to compute the matrix of the distances among each normalized segment couple, **DM**, whose elements are *DM_ij_* = *d*(*T̅*^(^*^i^*^)^, *T̅*^(^*^j^*^)^). At the same time, we compare the computed distance with a threshold, *d_th_*, representing the maximum acceptable distortion, due to compression (depending on the adopted dissimilarity metric, this threshold may take a different meaning; for instance, in the case of the infinity norm, it is the maximum relative deviation of the decompressed sample compared to the original one). When the distance between two segments *i* and *j* is lower than the threshold, we say that the two segments *match*. Hence, we can write the matching matrix MM, whose elements are *MM_ij_* = *I*(*DM_ij_* < *d_th_*), where *I*(·) is the indicator function.


**Algorithm 1** Matching-Basedmotif extraction algorithm.**Require:** DM, the precomputed distance matrix with *d*(·), a dissimilarity metric; *d_th_*, the threshold for the matching definition; *K_target_*, the maximum size for the Codebook. **for all**
*m*, *n*
**do**   *MM_mn_* ← *I*(*DM_mn_* < *d_th_*) **end for** *k* ← 1 **while** DM ≠ ∅ and *k* ≤ *K_target_*
**do**  **for all**
*m*
**do**    *MC_m_* ← ∑*_n_MM_mn_*  **end for**  *i* ← argmax*_m_*(*MC_m_*)  *X*(*k*) ← *T*^(^*^i^*^)^  delete the *l*-th rows and columns in DM and MM for which *MM_il_* = 1  *k* ← *k*+1 **end while** *K* ← *k*

**Figure 2.**
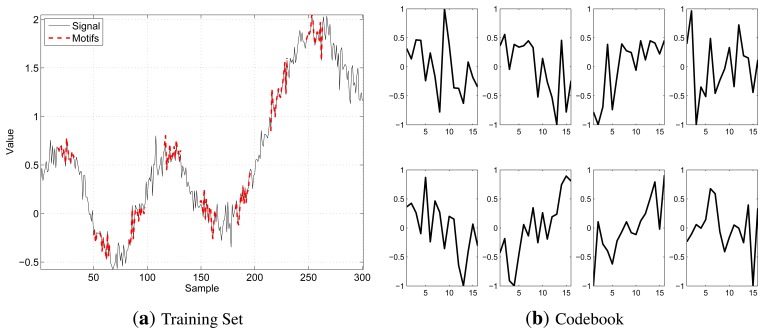
An example of the extraction procedure for *N* = 16, *K* = 8 and *b* = 8: (**a**) shows a given training set, highlighting, in bold red, the segments selected to form the Codebook; (**b**) represents the Codebook. Note that in the Codebook, the motif values range is [−1, 1].

As per our objective, the best segment to be included in the Codebook is the one obtaining the highest number of matchings and that is representative of all the segments it matches with, since their distance is lower than the threshold. Hence, our algorithm iterates, until either the training set is empty or the desired Codebook size, *K_target_*, is reached by selecting at each step the best segment and deleting it and all the segments that match with it from DM and MM. In case of ties, when two or more segments have the same number of matches, our algorithm picks that with the smallest average distance from the segments for which it is representative. The selected motifs will be referred to as *X*(*k*), *k* ∈ {1,…, *K*}. Figure 2 shows a training set example (on the left), highlighting, in bold red, those segments selected by the extraction algorithm to form the Codebook and the generated Codebook (on the right), for *K* = 8, *N* = 16 and *b* = 8. In the Codebook, the segments are stored in their normalized version, as they take values in [−1, 1].

Note that in the previous version of the algorithm we chose not to fix *d_th_*, but to let it vary as a function of the desired Codebook size. In this paper, instead, we preferred to have *d_th_* fixed to ease the comparison between RAZOR and other compression algorithms from the literature. However, this choice implies that, depending on the original signal variability, the desired Codebook size may be either higher or lower than the actual number of motifs needed for a complete representation of the training set. In turn, this translates in either a distortion higher than *d_th_* or a suboptimal usage of the Codebook size. In fact, since the compressed signal is sent by using motif indices, a motif belonging to a 16-element Codebook needs four bits to be sent; however, if the Codebook has only 9–15 motifs, its indices still need four bits, which, however, are used less efficiently.

Finally, we fixed the threshold *d_th_* = 16%, because, based on our results in [4], we found that too small a threshold would lead to a larger number of segments having the same number of matchings, thus forcing the algorithm to pick a large number of motifs at the beginning of the training set without selecting segments over the whole time series, while too large a threshold would lead to an increased number of matchings for each segment, thus forcing the algorithm to select a limited number of motifs; *d_th_* = 16% was found to be a good trade off value capable of building codebooks with *K* ≤ 64 (small enough) and a good distribution of the selected segments within the whole signal (large enough).

#### Motif representation

Finally, in order for the Codebook to maintain a limited size, we quantize the motifs using an alphabet of 2*^b^* symbols before saving them in the Codebook. Given our previous decompression procedure, we found that increasing *b* above eight provides minimal benefits, while using a *b* as low as four will further reduce the Codebook size, while, at the same time, maintaining good performance.

#### Multiple signal sources

Usually, in the IoT, multiple devices are in charge of delivering readings of the same phenomenon to a shared gateway. In this case, the description above holds with the following variation:

(12) $$\begin{array}{l} S_{n}(t)\in \mathbb{R},t\in [0,+\infty ),n\in N\\ S_{i,n}=Q(S_{n}(iT))\in A,i=1,\ldots ,M,n\in N \end{array}$$

which are the signal and the related quantized sample read by device *n* of the set 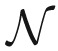 of the nodes of the network. Thus, the training set, *T*, becomes:

(13) $$T=\underset{n}{\cup }S_{i,n,}i=1,\ldots ,M,n\in N$$

where the ∪(·) operator stands for the concatenation of the different inputs. Moreover, the gateway must avoid creating a segment from samples belonging to different devices, because this would lead to physically infeasible signal shapes.

### 4.2. Motif Communication

As described in Section 3, IoT typical environments consist of many constrained devices and a few more powerful devices in charge of gathering data from the constrained devices and sending them towards the final destination. Due to the different computational capabilities, we designed RAZOR to perform the processing intensive operations (e.g., the motif extraction algorithm) at the gateway, while simple devices only need to use the Codebook computed by the gateway to compress their data.

The gateway, after receiving the needed amount of uncompressed data, runs the motif extraction algorithm, which eventually generates a Codebook that is representative for all the devices that contributed to the training set. For the signal we used to test RAZOR, we found that a few hundred samples (e.g., 300) were sufficient to discover the most significant patterns. However, this depends on the actual signal under analysis and must be dealt with carefully when applying the compression algorithm to unknown information sources.

A node will continue to send uncompressed data until the gateway distributes the Codebook to the network, but after its reception, each node is able to exploit the received information to compress *N* consecutive samples, sending the index of the best motif representing them. Note that these indices need log_2_(*K*) bits to be sent, but, since the Codebook contains normalized motifs, nodes have to also send offset and gain values along with the motif index to allow the receiver to decompress the signal properly.

Depending on the application accuracy requirement, it is possible to further quantize the offset and gain values with *b_O_* and *b_G_* bits, respectively. The quantized version of offset and gain are defined as *O̅* ∈ [min(*S*),max(*S*)] and *G̅*∈ [min(*G*),max(*G*)], respectively (min(*S*),max(*S*),min(*G*) and max(*G*) are computed on the training set by the gateway and sent along with the Codebook). As a result, the number of bits needed to send *N* consecutive samples is the sum of log_2_(*K*), *b_O_* = 8 and *b_G_* = 8 instead of the actual values of the samples that, for typical applications, may need up to 16 bits each, for a total of 16 *N* bits.

A final consideration about motif communications is related to packet losses. As in all compression techniques, each single packet carries a lot more information than uncompressed data. Although we are aware that packet losses can heavily impact RAZOR's performance, here we aimed at comparing RAZOR's effectiveness at the application layer with the state-of-the-art compressors and classificators for WSAN. Although a thorough study of RAZOR in multi-hop lossy networks and of how to improve its robustness is left as an interesting subject of further investigation, in this paper we provide some evaluations, where the effects of packet losses and multi-hop communications are assessed.

#### Motif selection

Since we are dealing with constrained devices, it is important to limit the computational complexity of the procedure applied to select which Motif to use to compress the signal. Here, for a given segment, *S*^(^*^i^*^)^, we compute its distance to each of the motifs in the Codebook and pick the motif *X̂*^(^*^i^*^)^ that minimizes that distance (note that we need to use the same dissimilarity metric used in the extraction phase in order to preserve the physical meaning of the distance). In symbols, this is:

(14) $${\hat{X}}^{\left(i\right)}=X_{R}(l),l=\underset{k}{\arg\min d}(S^{\left(i\right)}-X_{R}(k))$$

where *k*,*l* ∈ {1,…, *K*}, *i* ∈ 0,1,…,*M* − *N* + 1 and *X_R_*(*l*) ∈ ℝ is the de-normalized version of *X*(*l*), the *l*-th motif in the Codebook. Algorithm 2 provides the listing of the motif selection process.



**Algorithm 2** Motif selection algorithm.
**Require:**
*B*, the Codebook of the motifs; *S*^(^*^i^*^)^, the segment to be compressed; *d*(·), a dissimilarity metric; *d_th_*, the compression distortion threshold. *d_min_* ← ∞ 
$\bar{O}\leftarrow Q((1/N)\sum_{k=1}^{N}S_{i+k-1})$ *G̅* ← *Q*(*GAIN*(*S*^(^*^i^*^)^)) **for all**
*X*(*k*) ∈ *B*
**do**  *X_R_*(*k*) ← *X*(*k*)*G̅* + *O̅*  **if**
*d*(*S*^(^*^i^*^)^,*X_R_*(*k*)) < *d_min_*
**then**   *X̂*^(^*^i^*^)^ = *X_R_* (*k*)   *d_min_* = *d*(*S*^(^*^i^*^)^,*X_R_*(*k*))  **end if** **end for** Return *k*


Although a more thorough analysis on the computational cost of this operation will be provided in Section 5, we will give here some insight on the processing requirements. In order to find the best motif, the distance metric has to be computed *K* times. Hence, the more complex the evaluation of the distance and of the gain, the more computational demanding the process. However, since our Codebook is ordered according to the number of matchings obtained by each motif in the training set, it is more likely that the best Motif is one of the first in the Codebook. Hence, it is possible to stop the search as soon as the computed distance is lower than the given compression distortion threshold, *d_th_*. Note that stopping the algorithm before the search is finished decreases the computational complexity, but might not find the best motif in the Codebook, as just the first motif satisfying the requirement is picked.

Moreover, in order to provide a hardware-independent computational complexity evaluation, we note that our algorithm depends on two parameters: *N* and *K*. In particular, RAZOR's motif selection, in the worst case, needs to perform *N* distance computations between each of the *K* motifs in the dictionary and the segment under analysis. Considering a single segment, the complexity is equal to O (*KN*), and in order to compare RAZOR with other compress techniques, we need to consider the entire signals of length *M*. Thus, a signal of *M* samples is divided in segments of *N* samples; then, considering the complexity for a single segment, the total computational complexity is equal to O (*KM*) and does not depend on any network parameter. Table 1 reports the complexity for each of the techniques considered.

After the Motif selection phase, the node is able to send *k*, the index of *X̂*^(^*^i^*^)^, using log_2_(*K*) bits, *O̅*, the quantized value of the offset using *b_O_* bits, and *G̅*, the quantized value of the gain using *b_G_* bits. At the receiver side, the decompressed segment, *S_D_*, is computed as:

(15) $$S_{D}=X_{R}(k)=X(k)\bar{G}+\bar{O}$$

**Figure 3.**
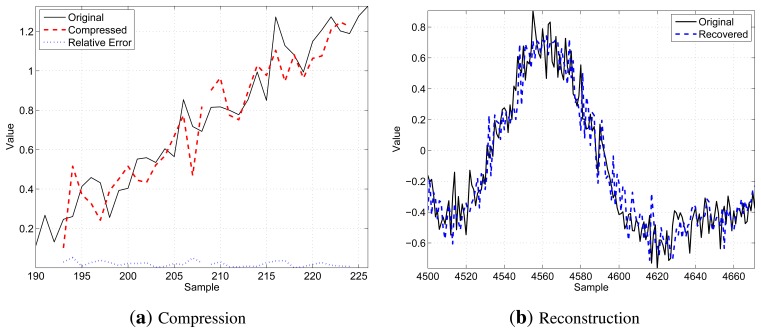
A qualitative example of the transmission procedure for *N* = 16, *K* = 8 and *b* = 8: (**a**) shows two consecutive segments (thin black solid line) of *N* samples, as well as the related compressed values (bold red dashed lines) and the relative compression error (thin blue dotted lines in the bottom part of the chart); (**b**) shows the result of the decompression operation comparing a longer segment of the original signal (thin black solid line) with the received data after the decompression (bold blue dashed line).

Figure 3 shows a qualitative example of the decompression procedure for *N* = 16, *K* = 8 and *b* = 8: on the left side, Figure 3a illustrates two consecutive segments (thin black solid line) of *N* samples, as well as the related compressed values (bold red dashed lines) and the relative compression error (thin blue dotted lines in the bottom part of the chart), while on the right side, Figure 3b shows the result of the decompression operation comparing a longer segment of the original signal (thin black solid line) with the received data after the decompression (bold blue dashed line). These figures have been chosen to provide graphical examples of what a motif looks like and how the re-constructed signal compares to the original one.

#### Motif prediction

After the Codebook has been computed, it is possible to perform an additional analysis, which will enhance RAZOR's performance. Since the training set is a realization of the physical phenomenon under study, it is possible to compress it with the Codebook and study the sequence of the best motif used for each segment.

In particular, we can count how many times a given motif, *X*(*l*), is used given that another motif, *X*(*k*), has been used for the previous segment. In such a way, we build the prediction matrix, PM, so that the element *PM_kl_* is the probability that *X*(*l*) is the best motif after *X*(*k*):

(16) $$PM_{kl}=\text{Prob}\{{\hat{X}}^{\left(i\right)}=X(l)|{\hat{X}}^{\left(i-N\right)}=X(k)\}$$

where *X̂*^(^*^i^*^−^*^N^*^)^ is the best motif at the previous, *i* − *N*-th step of the algorithm. This information can be exploited to order the motifs in the selection algorithm (see Section 4.2), so that they are tested in order of decreasing probability of having *X*(*l*) as the best motif given that *X*(*k*) was selected for the previous segment. Algorithm 3 provides the listing of the motif selection process with prediction.



**Algorithm 3** Motif selection algorithm with prediction.
**Require:**
*B*, the Codebook of the motifs; *S*^(^*^i^*^)^, the segment to be compressed; *d*(·), a dissimilarity metric; *d_th_*, the compression distortion threshold; PM, the prediction matrix; *X⌂*^(i−^*^N^*^)^, the previous best motif. *d_min_* ← ∞ 
$\bar{O}\leftarrow Q((1/N)\sum_{k=1}^{N}S_{i+k-1})$ *G̅* ← *Q*(*GAIN*(*S*^(^*^i^*^)^)) sort *B* according to the row of PM related to *X̅*^(^*^i^*^−^*^N^*^)^ **for all**
*X*(*k*) ∈ *B*
**do**  *X_R_*(*k*) ← *X*(*k*)*G̅* + *O̅*  **if**
*d*(*S*^(^*^i^*^)^,*X_R_*(*k*)) < *d_min_***then**   *X̂*^(^*^i^*^)^ = *X_R_* (*k*)   *d_min_* = *d*(*S*^(^*^i^*^)^,*X_R_*(*k*))  **end if**  **if**
*d_min_* ≤ *d_th_*
**then**   Break  **end if** **end for** Return *k*


Note that the benefits provided by this improvement are two-fold: on the one hand, it is possible to further reduce the number of bits for the representation of the compressed segment, and on the other hand, it is more likely that the selection algorithm stops before the end of the search operation.

The former advantage is justified by adding another bit to those sent by the compressing node. This bit is a flag, *F*, of size *b_F_* = 1 denoting whether or not the sent motif is the first, *F* = 1, according to the order suggested by PM, *i.e*., in other words, the *predicted* motif. If it is, it is possible not to send the index of the motif itself, because the receiver can infer it from PM; conversely, if the prediction is wrong, the motif index is sent, and the flag is set to zero, *F* = 0. Hence, the number of bits sent, *b_S_*, becomes:

(17) $$b_{S}=\{\begin{array}{ll} b_{F}+\log_{2}(K)+b_{O}+b_{G} & \text{if}\;F=0\\ b_{F}+b_{O}+b_{G} & \text{if}\;F=1 \end{array}$$

Although it is not assured that the predicted motif is the best, it is sufficient for this to happen with a frequency, *f*, higher than 1/log_2_(*K*) (e.g., for *K* = 8, the prediction needs to succeed once every three segments).

The latter benefit, instead, is motivated by observing that PM provides the marginal distribution of the best motif given the motif selected at the previous step. Hence, the selection algorithm is more likely to end sooner, because the most probable motifs are considered first. Note that this method is equivalent to encoding the Codebook using a Huffman code, where only the most probable motif is known and all the others are assumed equiprobable; we choose not to use a complete Huffman code for the Codebook, because that would have required a much longer training set than what we used.

#### Final considerations

Two final aspects are to be described in this section, before starting the evaluation of the solution: the numerical precision used within the online algorithm running on the nodes and the usage of different Codebooks to identify which physical phenomenon a given sensor is measuring.

The first topic has an impact on both the decompression distortion and the computational cost: in fact, the use of floating point numbers will dramatically increase the accuracy in the analysis phase, but has almost no impact on the decompression operation, since this process is dominated by the quantization of the offset and the gain. At the same time, imposing a distortion threshold, *d_th_*, makes it useless to obtain a representation of the decompressed motif more accurate than the threshold used during the extraction phase. Moreover, since floating point operations cost one or two orders of magnitude more than fixed point operations, the latter are to be preferred for constrained device implementation.

Finally, RAZOR, as introduced before, can be used to tell which physical phenomenon a given sensor is measuring. To do so, the simplest procedure is to obtain the Codebooks related to the variety of phenomena of interest for the considered application and, then, use each of the Codebooks to compress a given training set of the data produced by the sensor under analysis. Data is more likely to belong to the phenomenon whose Codebook obtains the smallest distortion.

Even when the signal classification should fail, our technique lets the system find the best Codebook from the known ones to compress and transmit the output of an unknown device, which is still a desirable feature.

## 5. Results

This section focuses on the evaluation of the proposed solution and its comparison with state-of-the-art techniques for both compression and classification. The section is split into four parts: Section 5.1 provides the definition of the evaluation metrics, as well as a few additional concepts used in the rest of the section for the evaluation; Section 5.2 evaluates our solution alone, analyzing the impact of the various setup parameters; Section 5.3 extends the previous evaluation comparing our solution against other signal processing techniques; an analysis of the performance in a real deployment scenario is provided in Section 5.4.

As to the datasets used in our evaluation, we considered three different databases:

1. The first dataset is obtained with a synthetic signal generator [24], configurable in terms of noise and correlation length. We let the correlation length vary between 20 and 100 samples, and we will refer to this set as the *SYN* dataset. This dataset has been used to test the compression capabilities of the three algorithms with different noise levels, *i.e*., changing the correlation length of the signal. EMMA has not been tested on this signal, because all the generated synthetic signals were likely to be classified in the same category, as they are intended to reproduce the same phenomenon.
2. The second dataset is also synthetic and is taken from the University of California Riverside (UCR) Time Series Classification/Clustering Homepage [25]: in particular, we tested our algorithms on the Synthetic Control dataset only, because it is the closest to IoT requirements. This dataset, referred to as *UCR*, has been used to compare RAZOR's and EMMA's classification performance. Instead, we could not use this set to test the compression performance, because it consists of fairly short sequences.
3. The last dataset that we used for testing is obtained from a real deployment, where we measured usual IoT readings, such as temperature, humidity and light. We refer to this dataset as *REAL*. We used this dataset to test RAZOR's compression performance on actual measurements, but we omit showing the comparison with the other algorithms for this dataset, because the obtained results were almost exactly the same as those obtained for the SYN dataset.

The algorithms have been studied using Matlab 2012. Each simulation has been performed varying each tunable parameter on a Sun Grid Engine (SGE) managed cluster with 100 CPUs on 16 racks with 16 GB RAM each. SGE has the purpose of scheduling the submitted simulations into different CPUs and to report the output of each simulation.

### 5.1. Evaluation Metrics

As in our previous work [4], the metrics considered to evaluate our technique are the compression rate, the signal distortion introduced as a consequence of the lossy compression and the classification accuracy. In addition, we will evaluate the processing cost and the energy expenditure on reference constrained hardware.

#### Compression

Although many definitions can be applied to quantify the compression effectiveness of an algorithm, we define *C* as the ratio of the compressed size to the original data size, which gives a simple formulation for deriving the number of bits to be sent. In its most basic formulation (see Section 4.2) and assuming that the uncompressed data are represented using 16 bits per sample, the compression rate is given by the following deterministic formula:

(18) $$C=(\log_{2}(K)+b_{O}+b_{G})/(16N)$$

where *K* is the number of motifs in the Codebook, *b_O_* and *b_G_* are the numbers of bits needed to represent the offset and the gain of the compressed segment, respectively, and *N* is the length of the segment in samples. The evaluation of the compression achieved using the prediction enhancement also depends on the success rate, *f*, of the motif prediction: in fact, in the case of a successful prediction, only one bit more than those for the gain and offset is needed, while in the negative case, all the information is to be sent, plus the additional bit needed for the prediction outcome. Hence, the compression rate becomes:

(19) $$C=[f(1+b_{O}+b_{G})+(1-f)(1+\log_{2}(K)+b_{O}+b_{G})]/(16N)$$

#### Distortion

In order to avoid confusion between the symbols used for errors and energy later on, we prefer to indicate the distance between the decompressed and the original signal samples as distortion. Furthermore, we will provide three different measures for the distortion: the maximum distortion, *D_M_*, the average distortion, *D_A_*, and the root mean square distortion, *D_R_*. The three quantities are defined as follows:

(20) $$\begin{array}{l} D_{M}=\max_{i}\left|S_{D,i}-S_{i}\right|/A,\\ D_{A}=(1/M)\sum_{i=1}^{M}\left|S_{D,i}-S_{i}\right|/A,\\ D_{R}=(1/A)\sqrt{(\sum_{i=1}^{M}{\left(S_{D,i}-S_{i}\right)}^{2})/M,} \end{array}$$

where *S_D,i_* and *S_i_* are the *i*-th samples of the decompressed and the original signals, respectively, *A* is the amplitude of the original signal and *M* is the length of the signal.

#### Complexity and energy consumption

When dealing with constrained devices, it is of paramount importance to maintain the computational complexity limited and, in particular, to ensure that the improvement provided by the technique does not require too much energy to be achieved.

For this reason, in Section 4.2, we have considered a hardware-independent metric (the complexity of the algorithm in terms of number of operations), which is useful for comparing different solutions, regardless of the specific device used. Here, in order to provide a more detailed assessment of the complexity and energy cost of our technique, we will consider two additional metrics that depend on the specific hardware used, namely, the number of CPU cycles needed to compress a sample, *P*, and the amount of energy spent to compress and transmit a sample, *E*. Finally, we will use as a quality indicator for a given technique the ratio, *R*, of the difference between the energy spent by the analyzed technique and the baseline energy expenditure (*i.e*., not compressing the signal) to the baseline expenditure itself. *R* > 0 translates into a proportional increase of the energy needed using the procedure, while, *vice versa*, *R* < 0 stands for a proportional energy saving.

The definitions of the aforementioned quantities are as follows: *P* is obtained as the ratio of the total number of CPU cycles needed for a given technique to process a signal, *C_CPU_*, to the number of samples of the signal, *M*; while *E* is calculated as the ratio of the sum of the number of bits sent times *E_RF_* (the energy needed to send a bit) and *C_CPU_* times the energy needed for a single CPU cycle, *E_CPU_*, divided by *M*. Finally, the baseline energy expenditure, *E_B_*, used to compute *R* is that of the telosb [3] wireless sensor architecture, because it is one of the most popular IoT architectures currently used by researchers. The three quantities can be summarized as follows:

(21) $$\begin{array}{l} P=C_{\text{CPU}}/M\\ E=(16ME_{RF}+C_{\text{CPU}}E_{\text{CPU}})/M\\ R=(E-E_{B})/E_{B} \end{array}$$

Telosb devices are equipped with a Texas Instrument (TI) CC2420 [26] radio transceiver, which spends *E_RF_* = 0.23*μ*J per transmitted bit and with a TI MSP430 [27] that has an average consumption of *E_CPU_* = 0.726 nJ per cycle. For every compression method, we have recorded the number of operations to process the original time series, accounting for the number of additions, subtractions, multiplications, divisions, powers, square roots, assignments, comparisons and absolute value operations. Thus, we have mapped these figures into the corresponding number of clock cycles, and we have subsequently mapped the latter into the corresponding energy expenditure: as a reference, we listed the number of processor cycles needed for each operation in Table 2 (the operator ≷ stands for comparisons between the two operands;, where we gave both fixed- and floating-point costs. Note that, in our experiments, we accounted for fixed-point operations for all the algorithms.

#### Classification accuracy

Our last evaluation metric is related to the classification performance. In order to compute it, we first have to define how the classification procedure is performed by our solution.

Our assumptions are that this procedure is performed by the gateway and that some different Codebooks related to a variety of physical phenomena against which to test a given signal are available.

In particular, the classification is performed in a similar fashion as the construction of the prediction matrix: in fact, after the reception of the needed number of uncompressed samples, the gateway tries to compress them according to all the Codebooks available and analyzes the resulting distortion. If the distortion due to compression exceeds a given threshold (note that this threshold is higher than that used in the motif extraction phase, since the system must tolerate some variability), the signal is assumed not to belong to the reference category. In case multiple Codebook distortions do not exceed the threshold, the signal is assumed to belong to that category whose Codebook obtained the smallest distortion.

To evaluate the accuracy of such classification, we take into account three quantities: *CC*, the correct classification frequency; *EC*, the erroneous classification frequency (a signal belonging to the category under examination is classified, instead, in another category); and *FP*, the false positive frequency (a signal classified in the category under examination belongs to another category).

### 5.2. Parameter Analysis

The first set of results is related to the selection of RAZOR's parameters. This selection has been made using the SYN dataset and spanning the whole parameter space. However, in what follows, we will study the impact of one parameter at a time in order to simplify the discussion.

We start our analysis by discussing the effectiveness of the prediction phase of the RAZOR algorith we evaluated RAZOR with prediction with respect to the standard version in terms of the energy sav percentage and the distortion loss for *N* = 16, …, 32 and *K* = 8, …, 32. The obtained results (reported here) showed that guessing the most probable motif given the motif chosen at the previous s can save about 10% of the energy expenditure, sacrificing less than 1.5% of the distortion.

**Figure 4.**
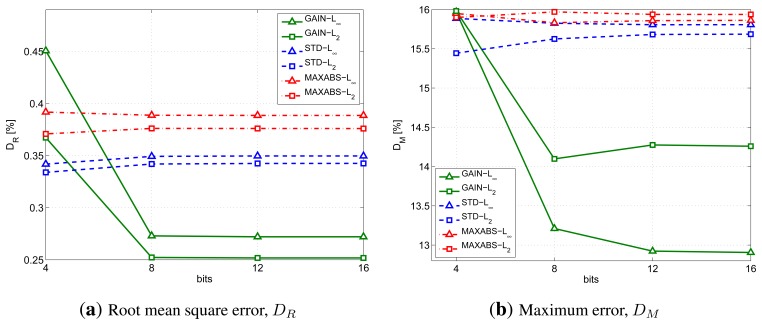
Analysis of the impact of the number of bits used on the compression distortion: (**a**) shows the RMSE of the different dissimilarity metric and gain combinations; while (**b**) provides the maximum error on the same combinations. The Codebook has been computed using *K* = 16 motifs of *N* = 20 samples.

Figure 4 shows the effect of the number of bits used on the compression distortion. In particular, Figure 4a provides the Root Mean Square Error (RMSE) for different dissimilarity metric and g combinations, while Figure 4b provides the maximum error. The Codebook has been computed us *K* = 16 motifs of *N* = 20 samples and *d_th_* = 16% of the original segment amplitude. In the figures, the GAIN, STD and MAXABS techniques are plotted with green solid lines, blue dashed lines a red dash-dotted lines, respectively. Instead, triangular and square markers represent the infinity and *L*2 norm, respectively.

The results show that it is not useful to increase the number of bits, *b*, beyond eight, because both distortion figures do not show proportional improvements, while the memory occupation of the Codebook increases linearly with the number of bits.

**Figure 5.**
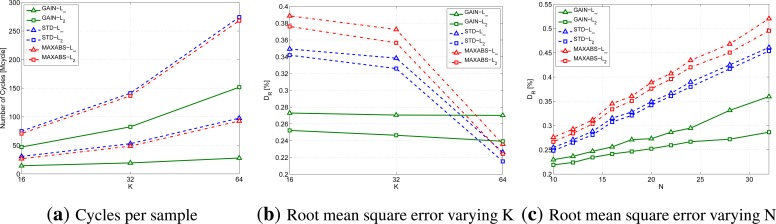
Analysis of the impact of the gain formula and of the norm used on the computational cost ((**a**), with *N* = 20 and varying *K*) and on the RMSE varying the number of motifs, *K* with *N* = 20 (**b**) or the motif length, *N*, with *K* = 16 (**c**). All graphs are plotted for *b* = 8.

To complete the analysis on the gain and the dissimilarity metrics, RAZOR needs to be tested varying *N* and *K*, while keeping fixed *b* = 8. Figure 5 provides the results we obtained to conclude the selection of the gain and the distance to be used.

In particular, Figure 5a shows the cycles per sample performed by the device CPU to compress the signal; this figure has been obtained for *K* = 16, 32 and 64 and *N* = 20. Note that the values picked for *K* are those that correspond to an integer value for log_2_(*K*). Similarly, Figure 5b provides the root mean square error using the same parameters.

Conversely, Figure 5c lets the motif length, *N*, vary while keeping fixed *K* = 16. The line styles and markers follow the same rules as above. (The number of cycles per sample is not included in the figures, because we found that it is not affected by *N*.)

From all these graphs, we can infer that, although it does not provide the best distortion, the combination of the GAIN formulation and the infinity norm obtains the best tradeoff between computational complexity and distortion. In what follows, the RAZOR algorithm will, therefore, always use the GAIN formulation, the infinity norm and eight bits for storing motifs in the Codebook.

As a final outcome of this parameter impact evaluation, we decided to use RAZOR with the prediction enhancement activated and fixing *N* = 20 and *K* = 16, because they grant good performance, while maintaining the size of the Codebook limited and below 500 bytes.

### 5.3. Comparison of the Techniques

In order to compare RAZOR against the other compression techniques, DCT and LTC, we tested the compression rate and the energy consumption ratio for the same distortion achieved by the different techniques. The signals we used for this test are those from the SYN dataset varying the correlation length from 20 to 200 samples.

**Figure 6.**
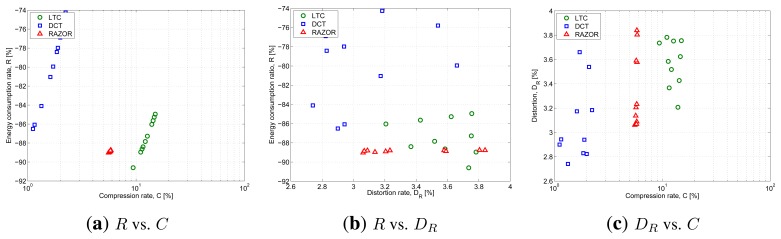
Performance comparison of three data compression solutions: RAZOR, in red with triangular markers, LTC, in green with round markers, and DCT, in blue with square markers. The three algorithms have been compared using three metrics: the compression rate, *C*, the energy consumption ratio, *R*, and the distortion, *D_R_*. The signals used in the graph are those of the SYN dataset, varying the correlation length from 20 to 200 samples.

Figure 6 plots the outcome of this evaluation and shows RAZOR, in red with triangular markers, LTC, in green with round markers, and DCT, in blue with square markers. The three figures explore three different trade-offs: Figure 6a plots the compression rate percentage, *C*, on the x-axis and the energy consumption percentage with respect to the baseline consumption (no compression), *R*, on the y-axis; Figure 6b plots the distortion, *D_R_*, on the x-axis and *R* on the y-axis; while Figure 6c plots *C* against *D_R_*.

The results show that RAZOR is able to save about 89% of the energy needed for transmitting uncompressed data, which is better than 86%–74% of DCT and on par with the 85–91% of LTC. In terms of compression rates, RAZOR shows intermediate performance (6%) between DCT (the best, with 3%) and LTC (the worst, with more than 8%). The sensitivity of RAZOR's compression and energy consumption performance to the correlation length is very weak (all points are very close to each other), whereas in the other two schemes a greater variability can be observed. The distortion performance is more dispersed for all three schemes, and while *R* and *C* present a strong degree of correlation with each other (see Figure 6a), *D_R_* is essentially uncorrelated with both *R* and *C* in all cases. For the sake of fairness, the computational cost of the three algorithms was computed assuming fixed-point operations for all of them.

Finally, we analyzed RAZOR's classification accuracy against EMMA [5]; to this end, we used the UCR dataset, averaging the results over different categories. Furthermore, we tried to set EMMA's parameters to offer a fair comparison with our solution. However, it must be said that EMMA has been designed to classify much longer time series than those we selected from the dataset and, therefore, cannot be expected to work very well in our case; this selection has been made to respect the computational constraints of our target devices.

**Figure 7.**
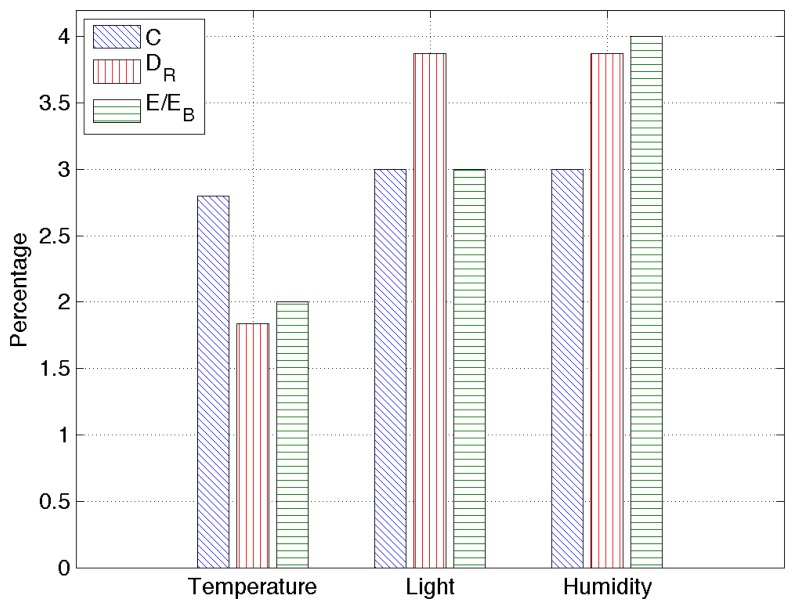
Classification accuracy comparison between RAZOR and Motifs through Matrix Approximation (EMMA): the CC, FP and EC bars report the correct classification, the false positive and erroneous classification ratios.

In Figure 7, we plotted the correct classification, false positive and erroneous classification ratios for the two algorithms, where blue bars on the left refer to RAZOR, while red bars on the right provide EMMA's results. These results show that RAZOR is able to obtain a very good correct classification ratio, whereas one of the state-of-the-art solutions for data mining fails when used with a very constrained configuration.

Based on the results in Figures 6 and 7, we can conclude that, even though RAZOR is neither the best compressor nor the best classifier, it is able to achieve good performance in both functionalities, while maintaining a low computational complexity, and can, therefore, be considered as a good lightweight tool for signal analysis and transmission in constrained environments. Its very low computational complexity reduces the energy consumption of constrained devices by about 90% with respect to the energy needed to send the measured data uncompressed. To strengthen this conclusion, in the final subsection, we will provide a short overview of RAZOR's results obtained on real data.

### 5.4. Real Scenario

The last set of results is obtained by applying RAZOR to a real dataset containing sensed information for temperature, humidity and light from 25 telosb nodes deployed in a building during two months. The training set has been computed over the data obtained during a single day, and all RAZOR parameters are set as described in the previous section.

**Figure 8.**
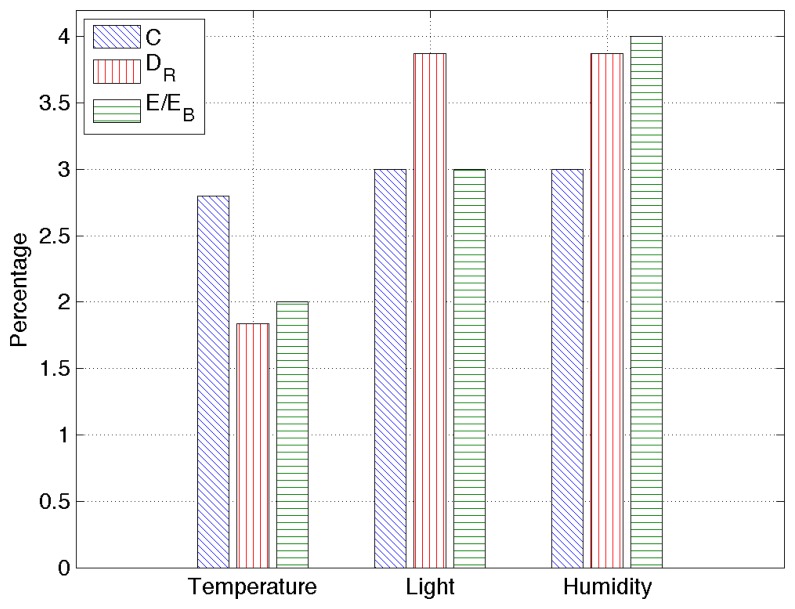
RAZOR's performance overview in a real scenario for different sensors: temperature, humidity and light. The performance figures are the compression rate percentage, *C*, the relative root mean square error, *D_R_*, and the percentage of energy used with respect to the energy needed to send uncompressed data, *E*/*E_B_*.

Figure 8 provides an overview of the obtained results: the compression rate percentage, *C*, the root mean square error, *D_R_*, and the percentage of energy used with respect to the energy needed to send uncompressed data, *E*/*E_B_*. Since all the percentages obtained here are very low, RAZOR's good performance is confirmed for real scenarios as well. In particular, our algorithm is capable of transmitting data over the network, consuming less than 10% of the energy needed to transmit the same data uncompressed, maintaining the root mean square error as low as a few percent.

## 6. Lossy and Multi-Hop Communications

So far, we only considered single hop communication and perfect channels (*i.e*., no packet loss). In this section, we will address both lossy channel and multi-hop communications in order to evaluate RAZOR's performance under more realistic environments.

First of all, we consider the impact of channel errors on the information loss, *I_L_*, computed as the ratio between the average number of bits lost over the total number of bits of the uncompressed signal, and on the communication efficiency, *η*, computed as the ratio between the average number of correct bits received over the total number of bits sent, under a non-perfect channel. In order to deal with different packet sizes, we computed our results starting from the bit error probability, *p_b_*, which is the ratio of the average number of erroneous bits over the total number of bits sent. From this and assuming errors to be independent and identically distributed (i.i.d.), we can derive the packet error probability, *p_p_* = 1 − (1 − *p_b_*)*^nb^*, where *n_b_* is the number of bits in a packet. (We leave the analysis of bursty channels for future work.)

Under these assumptions and with no retransmissions, transmitting the raw signal over a lossy channel with *p_b_* results in the following:

(22) $$I_{L}=\sum_{i=1}^{n_{p}}n_{b}p_{p}/(n_{b}n_{p})=p_{p}$$

where *n_p_* is the number of packets in the uncompressed signal. Instead, when using a compressor, the number of bits lost when a packet is corrupted amounts to the number of bits coded in the lost packet. Thus, Equation (22) becomes:

(23) $$I_{L}=\sum_{i=1}^{n_{p_{c}}}n_{b_{c}}p_{p}/(n_{b}n_{p})=n_{p_{c}}n_{b_{c}}p_{p}/(n_{b}n_{p})=\frac{n_{b}}{C}n_{p}C_{p_{p}}/(n_{b}n_{p})=p_{p}$$

where *n_bc_* = *n_b_*/*C* and *n_pc_* = *n_p_C* are the number of bits carried by a compressed packet and the number of compressed packets needed to send the whole uncompressed signal. Note that the information loss is exactly equal to the packet error rate for both the raw signal and the compressed signal, under our assumptions and if the packet size remains the same; however, the impact of information loss for compressors, such as DCT and LTC, depends on which packets are lost (in fact, in the case of DCT compression, the impact is much larger when the lost packet carries information about the low frequency coefficients of the transform, and in the LTC case, since a packet carries the parameters of the model for a large part of the signal, it is possible that a larger amount of bits of the original signal are lost as a consequence of losing a single packet.)

RAZOR's case is different, as a packet is smaller than a regular packet for WSAN; thus, RAZOR's packet error rate becomes *p_p_R__* = 1 − (1 − *p_b_*)*^n_b_R__^*, where *n_b_R__* < *n_b_* is the size in bits of a RAZOR's packet. Obtaining RAZOR's information loss rate is now straightforward:

(24) $$I_{L}=\sum_{i=1}^{n_{p_{c}}}n_{b_{c}}p_{p_{R}}/(n_{b}n_{p})=p_{p_{R}}<p_{p}$$

which amounts to RAZOR being more robust to channel errors, due to its smaller packet size.

Subsequently, if we account for a given number of retransmissions, *r*, the packet loss rate becomes $p_{p}(r)=p_{p}^{r+1}$, as a packet is only lost if all its transmissions are lost, for the raw and the general compressor case. For the RAZOR case, instead, given the smaller RAZOR's packet size, it is possible to piggyback retransmissions in subsequent communications by increasing the packet size: as an example, if the first packet is lost, the second packet can carry both the second and the retransmission of the first motif, thus saving bandwidth at the price of a slightly higher packet error rate and delay. The RAZOR packet error rate with *r* retransmission attempts becomes $p_{p_{R}}(r)=\prod_{i=1}^{r+1}\left(1-{\left(1-p_{b}\right)}^{in_{b_{R}}}\right)$. Now, it is possible to compute the information loss given any number of retransmissions for both the raw and RAZOR's case, which is equal to *p_p_*(*r*) and to *p_p_R__*(*r*), respectively.

In order to evaluate *η*, we need to recompute the average number of bits sent, which, in the raw case, is $n_{S}(r)=n_{b}\sum_{i=0}^{r}p_{p}(i)=n_{b}\frac{1-p_{p(i+1)}}{1-p_{p}}$, while for RAZOR it is slightly more complex, due to the increasing size of packets, and becomes $n_{S_{R}}(r)=\sum_{i=0}^{r}n_{b_{R}}(i+1)p_{p_{R}}(i)$. Finally, *η* is obtained as a function of the number of retransmissions as:

(25) $$\eta (r)=\frac{1-I_{L}(r)}{n_{S}(r)}$$

Finally, note that the compression rate, *C*, changes when retransmissions are accounted for, and it becomes *C*(*r*) *= n_S_*(*r*)/(*n_b_n_p_*), which translates in the ratio between the average number of bits sent over the size of the uncompressed signal in bits.

**Figure 9.**
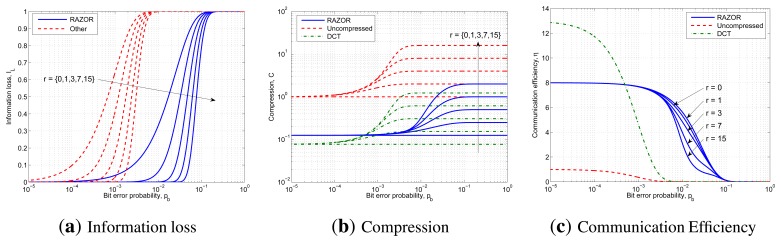
Performance evaluation in lossy channels varying the bit error probability of RAZOR (blue solid line), uncompressed data (red dashed line) and DCT (green dash-dotted line). RAZOR has been compared using three metrics: information loss, *I_L_*, compression, *C*, and communication efficiency, *η*. For each chart, we considered a number of retransmissions *r* ∈ {0, 1, 3, 7,15}.

Figure 9 shows the results of the previous analysis drawing with blue solid lines, red dashed lines and green dash-dotted lines the curves related to RAZOR, the uncompressed signal and DCT compression, respectively. In addition, the effect of the increasing number of retransmissions is directly annotated in the figures. In particular, Figure 9a, Figure 9b and Figure 9c plot the information loss, the compression and the efficiency as a function of *p_b_* ∈ [10^−5^,1].

Figure 9a does not show any line for the DCT, as this technique offers neither improvement nor degradation in terms of *I_L_* over the uncompressed technique, as is evident from Equation (23). Instead, due to its reduced packet size, RAZOR outperforms the other solutions by two orders of magnitude, as it is able to deliver almost all the information, even for *p_b_* = 10^−2^ when at least three retransmissions are allowed.

Figure 9b, which depicts the compression achievable if *r* = {0,1,3,7,15} retransmissions are allowed, demonstrates that DCT outperforms RAZOR almost everywhere, except in the central region of *p_b_*, thanks to its highest compression factor.

Finally, Figure 9c, which illustrates the efficiency of the three solutions, shows that DCT is the best compressor when the information loss is negligible, while RAZOR is clearly the preferable solution when *p_b_* > 10^−3^. Moreover, in terms of communication efficiency, *r* has no effect for both DCT and uncompressed transmission, while we noted that, for RAZOR, only a single retransmission improves the communication efficiency; thus, choosing the correct value for *r* is a trade off between robustness (high *r*) and energy consumption (low *r*).

For what concerns multi-hop communications, we analyzed the energy consumption figures for RAZOR, DCT compression and RIDA. For what concerns RAZOR and DCT, we computed the energy expenditure as the sum of the single hop consumption plus the energy needed to forward either the motifs (RAZOR) or the coefficients (DCT) from when they are computed in the network to the gateway. Instead RIDA, which is a hierarchical solution [22], needs first to compress the signal locally within each cluster and, subsequently, to send the computed coefficients to the gateway.

**Figure 10.**
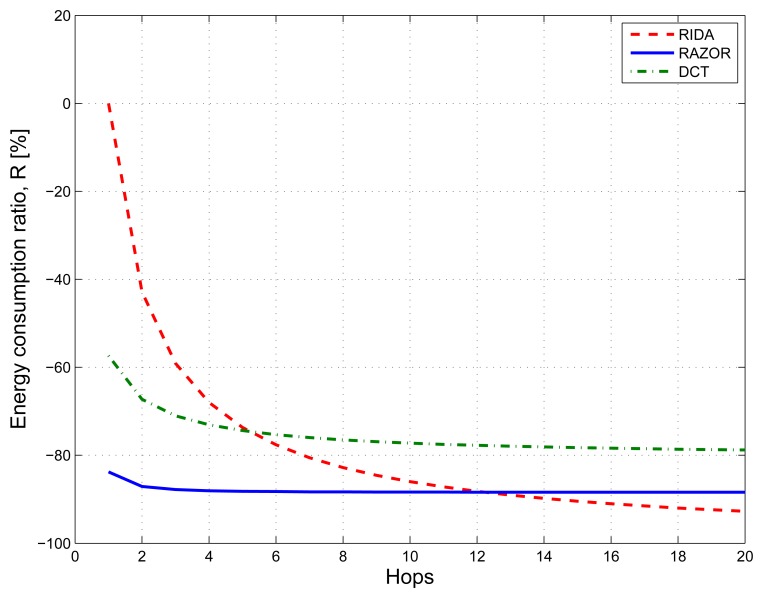
Performance comparison of three data compression solutions: RAZOR (blue solid line), DCT (green dash-dotted line) and RIDA (red dashed line). The three algorithms are compared in terms of the energy consumption ratio, *R*, varying the number of hops of the network.

Figure 10 shows with blue solid, red dashed and green dash-dotted lines the results for RAZOR, RIDA and DCT, respectively. While RAZOR and DCT have almost the same behavior with RAZOR outperforming DCT, thanks to its lower computational complexity, RIDA has a very high energy consumption (as high as what is needed to send the uncompressed signal) in small networks, but shows a rapid improvement as the network size increases, outperforming both DCT and RAZOR when the number of hops is larger than 12.

## 7. Conclusions

In this paper, we presented RAZOR, a novel lightweight algorithm for data compression and classification targeting constrained devices, such as those that will be encountered in the Internet of Things.

The main concept driving the design of our solution is derived from vector quantization and pattern recognition techniques: in fact, RAZOR works by first creating a Codebook at the gateway from a given training set of uncompressed data and, subsequently, distributing this Codebook to constrained devices to compress the following readings.

The Codebook is then used again by the gateway to interpret the compressed data received from the nodes. In addition, the gateway, or some other computationally powerful devices, can use different Codebooks to classify data produced by an unknown source.

In order to obtain the final definition of the RAZOR algorithm, we started from the analysis of our previous work, and we complemented it by considering different normalization formulas for data segments, different dissimilarity functions for the motif extraction algorithm and a novel technique to predict the motif to be transmitted, which is able to provide further improvements in terms of the compression rate.

We ran a thorough evaluation campaign to select RAZOR's best configuration and to compare it against state-of-the-art signal processing solutions, showing that our algorithm is able to obtain performance similar to that of the most relevant competitors in terms of both compression and classification, when used on constrained devices. In addition, we studied the impact of multi-hop and lossy communication on RAZOR performance, showing that it outperforms standard compressors when the error impact is not negligible, and can be preferable to a more complex hierarchical solution, when the network size is smaller than 12 hops.

Thus, RAZOR is a very good candidate for developing a versatile signal processing tool to be used in the IoT both to reduce the communication overhead when transmitting known signals and to classify unknown information sources.

Our future research will be focused on exploiting RAZOR to develop a data-based ontology capable of classifying signals depending on the actual data streams and to use the classification information in order to optimize communication in the constrained part of the network.

## Figures and Tables

**Table 1.** Computational complexity. DCT, Discrete Cosine Transform; LTC, Lightweight Temporal Compression (LTC); Robust Information-Driven Data Compression Architecture (RIDA).

**RAZOR**	**DCT**	**LTC**	**RIDA**
O(*KM*)	O(*MlogM*)	O(*M*)	O(*M*)

**Table 2.** Telosb operation costs.

	*a* + *b*	*a* − *b*	*ab*	*a*/*b*	*a^b^*	$\sqrt{a}$	*a* ← *b*	*a* ≷ *b*	|*a*|
Fixed point	4	4	15	23	15	250	1	4	4
Floating point	184	187	395	405	395	720	10	37	184
